# Interaction of MOPS buffer with glass–ceramic scaffold: Effect of (PO_4_)^3−^ ions in SBF on kinetics and morphology of formatted hydroxyapatite

**DOI:** 10.1002/jbm.b.34530

**Published:** 2019-12-16

**Authors:** Diana Horkavcová, Dana Rohanová, Adam Stříbny, Katharina Schuhladen, Aldo Roberto Boccaccini, Petr Bezdička

**Affiliations:** ^1^ Department of Glass and Ceramics, Faculty of Chemical Technology University of Chemistry and Technology Prague Prague Czech Republic; ^2^ Department of Materials Science and Engineering Institute of Biomaterials, University of Erlangen‐Nuremberg Erlangen Germany; ^3^ Institute of Inorganic Chemistry of the ASCR, v.v.i Husinec‐Řež Czech Republic

**Keywords:** bioglass®, glass‐ceramics scaffold, in vitro test, MOPS buffer, simulated body fluid

## Abstract

The international standard ISO 23317:2014 for the in vitro testing of inorganic biomaterials in simulated body fluid (SBF) uses TRIS buffer to maintain neutral pH. In our previous papers, we investigated the interaction of a glass–ceramic scaffold with TRIS and HEPES buffers. Both of them speeded up glass–ceramic dissolution and hydroxyapatite (HAp) precipitation, thereby demonstrating their unsuitability for the in vitro testing of highly reactive biomaterials. In this article, we tested MOPS buffer (3‐[N‐morpholino] propanesulfonic acid), another amino acid from the group of “Goods buffers”. A highly reactive glass–ceramic scaffold (derived from Bioglass®) was exposed to SBF under static–dynamic conditions for 13/15 days. The kinetics and morphology of the newly precipitated HAp were studied using two different concentrations of (PO_4_)^3−^ ions in SBF. The pH value and the Si^IV^, Ca^2+^, and (PO_4_)^3−^ concentrations in the SBF leachate samples were measured every day (AAS, spectrophotometry). The glass–ceramic scaffold was monitored by SEM/EDS, XRD, WD‐XRF, and BET before and after 1, 3, 7, 11, and 13/15 days of exposure. As in the case of TRIS and HEPES, the preferential dissolution of the glass–ceramic crystalline phase (Combeite) was observed, but less intensively. The lower concentration of (PO_4_)^3−^ ions slowed down the kinetics of HAp precipitation, thereby causing the disintegration of the scaffold structure. This phenomenon shows that the HAp phase was predominately generated by the presence of (PO_4_)^3−^ ions in the SBF, not in the glass–ceramic material. Irrespective of this, MOPS buffer is not suitable for the maintenance of pH in SBF.

## 1 INTRODUCTION

In accordance with ISO 23317:2014 (International Organization for Standardization, 2014), newly developed biomaterials intended for bone replacement must be tested in vitro with simulated body fluid (SBF). SBF models the inorganic part of blood plasma, which is supersaturated with respect to hydroxyapatite (HAp). Because of the need to suppress spontaneous HAp precipitation, TRIS buffer is used to maintain neutral pH for up to 4 weeks. However, there is some doubt about the stability of SBF in the presence of such a buffer.

In our previous papers, we reported the use of TRIS and HEPES buffers for the in vitro testing of an inorganic glass–ceramic scaffold (Rohanová et al., 2011, 2018). Both buffers interacted with a highly reactive material, a glass–ceramic scaffold derived from 45S5 Bioglass®. Moreover, they were not able to maintain the pH of SBF at a neutral level when the material released higher concentrations of Na^+^ ions (Rohanová et al., 2011, 2018). In fact, the presence of the buffers in SBF more than doubled the rate of glass–ceramic dissolution and enhanced HAp crystallization. We hypothesized that this was due to calcium bonding to buffers from SBF and/or the material. Bastos, Platt, Andrade, and Soares (2008) confirmed this theoretically by showing that TRIS/BisTRIS buffers affect Ca^2+^ and (PO_4_)^3−^ ion activities with an intensity‐dependent on buffer‐type and pH range. The formed Ca(TRIS)^2+^ species modified the free Ca content in SBF and the Ca(free)/P ratio influenced the stoichiometry of the precipitated phosphate. Further supporting our theory that the Ca^2+^ ions bond to the amino acids to form a soluble complex compound (Pietrzyňska & Voelkel, 2017). Altura, Carella, and Altura (1980) demonstrated that the TRIS, HEPES and 3‐(N‐morpholino) propanesulfonic acid (MOPS) buffers act directly on calcium ion exchange in the vascular smooth muscles of isolated rat aorta and portal vein.

The used buffer (weak electrolyte) plays a specific role also in a biological system (de Carvalho Dias, Aboud Barbugli, & Vergani, 2016; Gupta, Chen, & Lee, 2015; Salis & Monduzzi, 2016; Taha & Lee, 2010). Buffers ions compete with strong electrolytes for selective adsorption at the protein charged surface (Salis & Monduzzi, 2016). Taha and Lee (2010) conclude that many experiments have failed because of the imperfections of the buffers employ. They studied the ionic interaction from volumetric investigation, for example, MOPS in aqueous solutions of NaCl or KCl.

Not only ionic interactions in the biological system (de Carvalho Dias et al., 2016) but also the kinetics of calcium phosphate precipitation (biomineralization) could be affected by a choice of the buffer system. Various authors have attempted to explain the kinetics of HAp precipitation, probably in solutions without the TRIS buffer. According to van Kemenade and de Bruyn (1987), who studied the formation of different calcium phosphates (DCPD, OCP, HAp, and ACP) as a function of pH and supersaturation, the formation of HAp was found to be preceded by one or more precursors in agreement with the Oswald rule of stage. Homogenous formation of HAp at low concentrations was never observed. Moreno, Zahradnik, Glazmann, and Hwu (1977) studied the kinetics of HAp precipitation by seeding dilute supersaturated solutions with well‐characterized HA crystals. In a solution with an initial degree of supersaturation comparable to that in human serum, they found that the precipitation rates were related to the thermodynamic driving force (degree of supersaturation with respect to HAp) and not to solution composition. Bastos et al. (2008) designed “simplified SBF” (without TRIS buffer), which has a much higher concentration of HCO_3_^−^ ions (90 mM) than blood plasma. However, we investigated (Rohanová et al., 2014) that the interaction of our glass–ceramic scaffold with a nonbuffered cell culture medium (DMEM) with a higher concentration of HCO_3_^−^ ions (44 mM) leaded to the formation of calcium carbonate (CaCO_3_) and amorphous calcium phosphate phase (ACP) except HAp. This raises the question of whether “simplified SBF” is supersaturated with HAp or with CaCO_3_. Kim, Miyaji, Kokubo, Ohtsuki, and Nakamura (1995) studied the contribution of the P_2_O_5_ to the SBF supersaturation exposed in acellular SBF with the TRIS buffer. They observed a little difference in the rates of ion dissolution and of apatite formation between Bioglass 45S5 and P_2_O_5_ free Na_2_O‐CaO‐SiO_2_ glass and confirmed bioactivity of P_2_O_5_ free glasses. Thus, it seems, the P_2_O_5_ in Bioglass has insignificant contribution to the SBF supersaturation when the TRIS buffer was used. As implies from our recherché, the “bioactivity” studies could be strongly affected by the presence of the buffer both in SBF and other solutions supersaturated towards to HA.

In this study, we continue our investigation into the use of Good's buffers for the in vitro testing of inorganic glass–ceramic scaffolds, this time using MOPS buffer (Good et al., 1966). In order to compare it with our previously tested TRIS and HEPES buffers and to further understanding of the kinetics of HAp precipitation in the presence of MOPS. SBF for the testing was prepared according to ISO 23317:2014, except that of course MOPS replaced TRIS. A glass–ceramic scaffold derived from 45S5 Bioglass® was chosen for the testing. The interaction of the tested material with MOPS and influence of the kinetics of HAp formation were observed in two solutions: simulated body fluid (SBF + MOPS) and SBF with a reduced concentration of (PO_4_)^3−^ ions (SBF 70P + MOPS).

## 2 MATERIALS AND METHODS

### 2.1 Materials

The scaffolds were prepared from 45S5 Bioglass® powder by the foam replica method following the procedure described by Chen, Thompson, and Boccaccini (2006). The scaffolds were rectangular in shape (10 × 5 × 5 mm). All scaffolds exhibited porosity of ~90%, which was determined by the measurement of their mass and dimensions. The porosity was then calculated by equation described by Chen et al. (2006). A slurry for the impregnation of the sacrificial polyurethane foams was prepared by mixing glass particles with an aqueous solution of PDLLA (poly lactic l‐d acid). After drying, the porous precursor was sintered at 1,100°C for 5 hr. Partial crystallization of the glass occurred upon heat‐treatment. Bioglass® based glass–ceramic scaffolds fabricated by the foam replica technique exhibit one advantage for in vitro tests: after the thermal exposure to densify the struts, a main crystalline phase (Na_2_O∙2CaO∙3SiO_2_) and a minor phase (CaO∙SiO_2_) usually develop, which in the ideal case (100% crystallization) should consume all CaO present in Bioglass®. The residual glass phase should therefore contain the entire quantity of P_2_O_5_. Table 1 shows the composition of the starting 45S5 Bioglass® powder and of the main phases of the scaffolds investigated.

**Table 1:** Compositions of 45S5 Bioglass® and individual phases of the scaffold (wt%)

Oxide	45S5 BG (100 wt%)	Na_2_O∙2CaO∙SiO_2_ (77.4 wt% of scaffold)^a^	Residual glass phase (22.6% of scaffold)
SiO_2_	45. 0	50. 9	24. 8
Na_2_O	24. 5	17. 4	48. 5
CaO	24. 5	31. 7	–
P_2_O_5_	6. 0	–	26. 5

a Crystalline phase CaO∙SiO_2_ is included.

### 2.2 Solutions for in vitro test

In this study, the behavior of the glass–ceramic scaffold was investigated by exposing the materials to two types of modified solutions: (a) solution containing inorganic components similar to blood plasma in combination with a buffer labeled as SBF + MOPS, (b) SBF solution with low concentrations of (PO_4_)^3−^ ions labeled as SBF 70P + MOPS. Both solutions were prepared from the reagents: KCl, NaCl, NaHCO_3_, MgSO_4_, CaCl_2_, KH_2_PO_4_, and MOPS buffer concentration in the solution was 0.0375 mol dm^−3^. The ion composition of the solutions is shown in Table 2.

**Table 2:** Ion composition (mmol dm^−3^) of SBF + MOPS and SBF 70P + MOPS

	Na^+^	K^+^	Ca^2+^	Mg^2+^	Cl^−^	HCO_3_^−^	HPO_4_^2−^	SO_4_^2−^
SBF + MOPS	142.0	5.0	2.5	1.0	131.0	4.2	1.0	0.5
SBF 70P + MOPS	142.0	5.0	2.5	1.0	131.0	4.2	0.7	0.5

### 2.3 Static–dynamic conditions of in vitro test

The weight of glass–ceramic scaffold samples was 0.050 ± 0.005 g and they were placed in platinum spiral and immersed separately in plastic bottles with 50 ml of both types of solutions. The sample bottles were put into a biological thermostat at a temperature of 36.5°C. The solutions were replaced every 24 hr.

### 2.4 Leachate analysis

#### 2.4.1 *Atomic absorption spectrophotometry*

The concentrations of calcium and silicon in the leachate after glass–ceramics exposure were measured by means of atomic absorption spectrophotometry on SpectrAA 880 made by VARIAN. The flame used for atomization was acetylene–N_2_O. Calcium concentrations were measured at λ = 422.7 nm. Silicon concentrations were measured at λ = 251.6 nm. The flame used for atomization was acetylene–N_2_O.

#### 2.4.2 *Spectrophotometry*

The concentrations of (PO_4_)^3−^ ions in the leachates were determined at λ = 830 nm with the UV–Visible UV 1601 spectrophotometer, in conformity with ČSN 83 05 40.

#### 2.4.3 *pH measurement*

The pH values of both types of leachates were measured at 32.5–33.5°C using an inoLab pH meter (made in Germany) with a combined glass electrode.

### 2.5 Analysis of the glass–ceramic scaffold

#### 2.5.1 *Scanning electron microscopy/energy‐dispersive spectroscopy (SEM/EDS)*

The sample surface morphology was inspected by an Hitachi S‐4700 scanning electron microscope equipped with an energy‐dispersive spectroscopy analyzer (NORAN D‐6823) with SDD (Silicon Drifted Detector) using the acceleration potential of 15 kV. Samples were sputtered by Au/Pd layer for 100–120 s.

#### 2.5.2 *X‐ray powder diffraction analysis*

The glass–ceramic samples were ground in an agate mortar in a suspension with cyclohexane. The suspension was then put on a mylar film and placed into transmission sample holder. Diffraction patterns were collected with a PANalytical X'Pert PRO diffractometer equipped with a conventional X‐ray tube (Cu *K*α radiation, 40 kV, 30 mA, point focus) and a position‐sensitive detector PIXcel with an anti‐scatter shield. X‐ray patterns were measured in the range of 10 to 100° 2θ with step of 0.0131° and 200 s counting per step. Qualitative analysis was performed with the HighScorePlus software package (PANalytical, the Netherlands, version 2.2.5), Diffrac‐Plus software package (Bruker AXS, Germany, version 8.0) and JCPDS PDF‐2 database, International Centre for Diffraction Data (Newtown Square, PA release 54, 2004).

#### 2.5.3 *WD‐XRF*

Sequential wavelength dispersive X‐ray spectrometer Perform'X made by Thermo SCIENTIFIC was used for the X‐ray fluorescent analysis. It was equipped with an X‐ray lamp with an Rh anode type 4GN and a 50 μm thick Be end‐window. Intensities of all the spectral lines of elements were measured in vacuum with the OXAS software. Combinations of setups of the generator–crystal collimator–detectors were optimized for 82 measured elements for the times of 10 − 6 s for each element. The obtained intensities were processed by the UNIQUANT 5 software without the necessity to measure standards. The analyzed powder samples were compressed into tablets 5 mm thick with diameter of 40 mm without any binder. The measuring time of one sample was approximately 15 min.

#### 2.5.4 *B.E.T. measurement*

The specific surface area of glass–ceramic scaffolds was measured by the B.E.T. method with an ASAP 2020, Micrometrics device using the nitrogen at the temperature 77 K for 2 hr.

## 3 RESULTS

### 3.1 Analysis of SBF leachates

**Figure 1:**
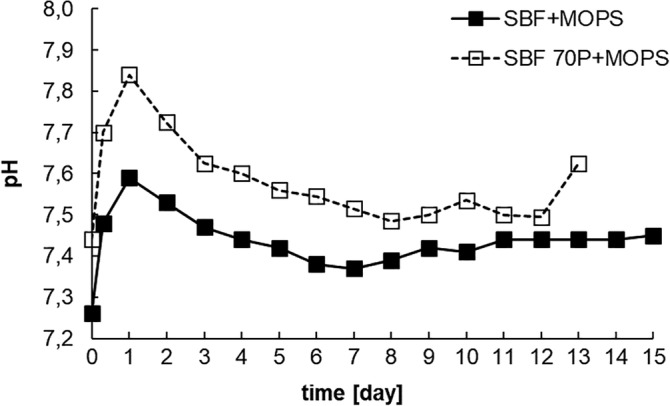
pH values in SBF + MOPS and SBF 70P + MOPS during interaction with scaffold (static–dynamic conditions of in vitro test)

As with the TRIS and HEPES buffers previously tested (Rohanová et al., 2011, 2018), the MOPS buffer did not maintain neutral pH during the testing of the glass–ceramic scaffold (Figure 1). In fact, in SBF 70P + MOPS the pH increased to an even higher alkaline level. The pH values show that the glass–ceramic scaffold interacted with each solution immediately after its submersion.

**Figure 2:**
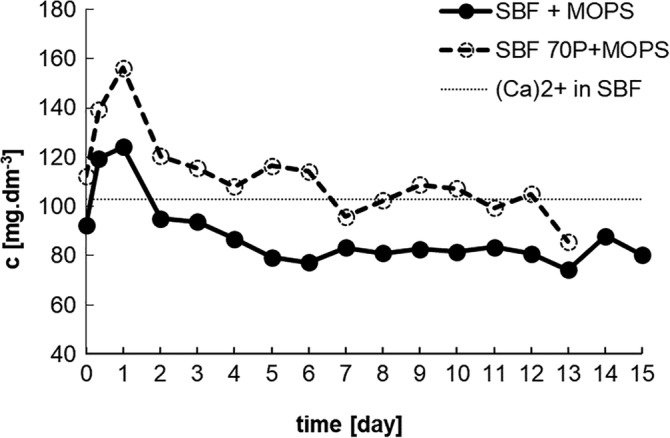
Concentration of Ca^2+^ ions in SBF + MOPS and SBF 70P + MOPS during interaction with scaffold (dotted line is original value of Ca2+ ions in SBF)

In both solutions, this immediate increase in pH corresponded with a dramatic increase in the concentration of the Ca^2+^ ions. After the second day, the concentration of Ca^2+^ ions decreased in and stabilized to around its original value (90 and 105 mg dm^−3^) in SBF + MOPS, respectively, SBF 70P + MOPS (Figure 2).

**Figure 3:**
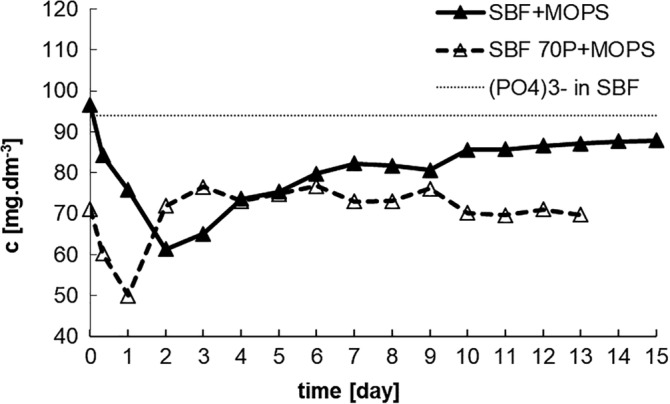
Concentration of (PO_4_)^3−^ ions in SBF + MOPS and SBF 70P + MOPS during interaction with scaffold (dotted line are original values of (PO_4_)^3−^ ions in SBF + MOPS and SBF 70P + MOPS)

In both solutions, there was a rapid decrease in the (PO_4_)^3−^ concentration as early as the first day. After the second day, the (PO_4_)^3−^ concentration in SBF + MOPS increased and stabilized on Day 6 reaching 80–87 mg dm^−3^. As in the case of the Ca^2+^ ions, but in this instance after just 1 day, in SBF 70P + MOPS, the (PO_4_)^3−^ concentration returned to and stabilized at around its original value (70 mg dm^−3^; Figure 3).

**Figure 4:**
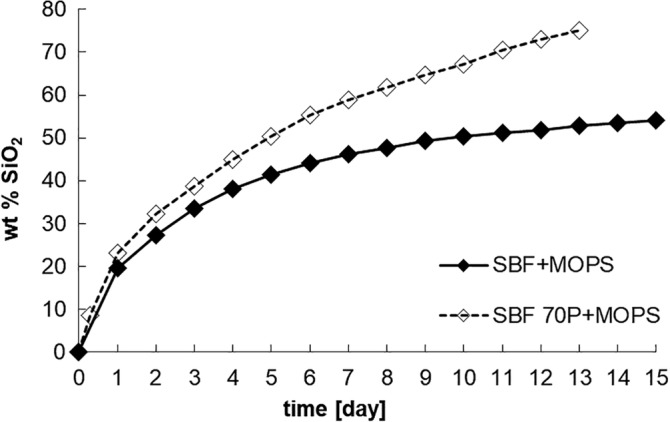
Cumulative dissolution of SiO_2_ in SBF + MOPS and SBF 70P + MOPS as calculated from material balance of leached Si (calculation see section 3)

The presence of Si (calculated as SiO_2_) in the leachates indicated the dissolution of the scaffold. During the in vitro tests, more than half of the original amount of SiO_2_ (54 wt%) was released from SBF + MOPS and around 75 wt% from SBF 70P + MOPS. By the end of Day 13, the scaffold had completely dissolved (Figure 4) in SBF 70P + MOPS.

### 3.2 Analysis of glass–ceramic scaffolds

**Figure 5:**
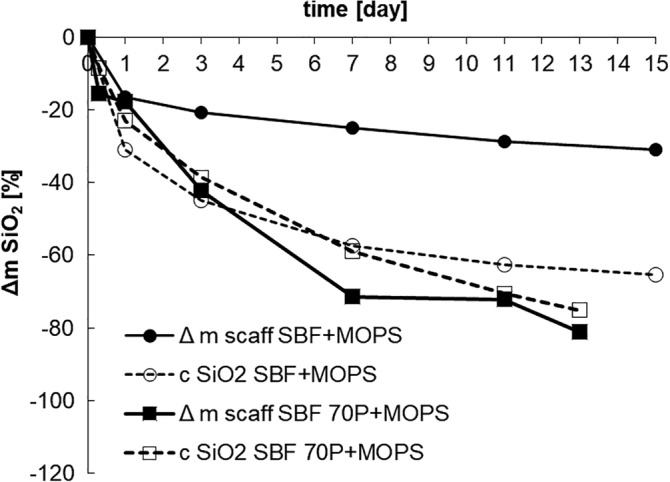
Decreases of scaffold weight in SBF + MOPS and SBF 70P + MOPS compare to the theoretical weight calculated from the Si released into solution during in vitro test

The weight of the scaffolds was measured before and after interaction (real weight), as well as being determined from the amount of SiO_2_ released into the leachate (theoretical weight). Both weights showed a significant difference between supersaturated SBF + MOPS and undersaturated SBF 70P + MOPS. In the case of SBF + MOPS, the theoretical weight of the scaffold differed significantly from the real weight. This difference reflects the weight of the newly formed Ca‐P phase. By the end of the test (Day 15) HAp represented up to 30 wt% of the original scaffold weight. However, when the SBF solution was undersaturated (in SBF 70P + MOPS), the theoretical weight was equal to the scaffold real weight, which suggests the negligible formation of the Ca‐P phase. Glass–ceramic scaffold did not release a sufficient amount of (PO_4_)^3−^ ions in solution to bond its structure with the Ca‐P phase. After 13 days, the scaffold was completely dissolved (Figure 5), which is in coincidence with solution analysis.

**Figure 6:**
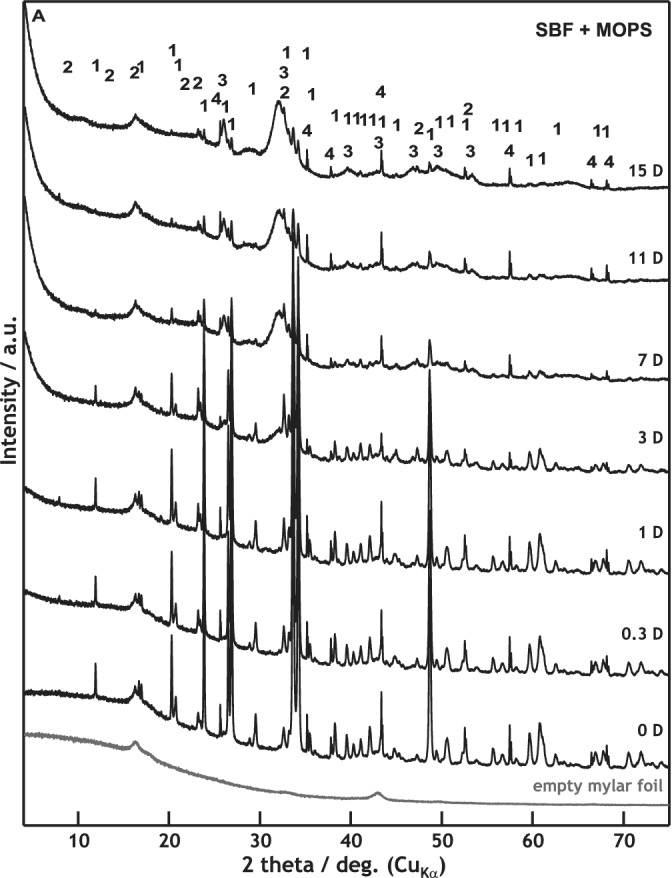
XRD patterns of scaffold samples before and after 0.3, 1, 3, 7, 11, and 15 days of interaction with SBF + MOPS

**Figure 7:**
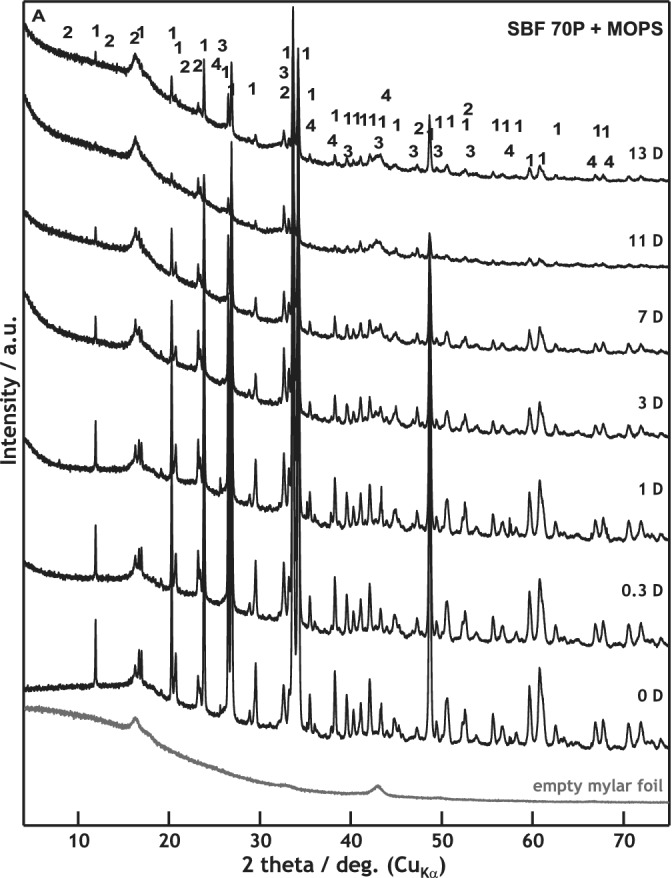
XRD patterns of scaffold samples before and after 0.3, 1, 3, 7, 11, and 13 days of interaction with SBF 70P + MOPS (scaffold was nearly dissolved after Day 13)

The XRD and XRF measurements provided a detailed view of the phase formation and dissolution. According to XRD, the dissolution of the major crystalline phase Combeite (Na_2_O∙2CaO∙3SiO_2_) started no later than 8 hr after submersion in the SBF + MOPS solution. The crystalline phases of the glass–ceramic scaffold (major as well as minor—CaO∙SiO_2_ and NaCaPO_4_) were completely dissolved after 15 days. The formation of the crystalline HAp started on the third day of in vitro testing (Figure 6). The major crystalline phase dissolved more slowly in the undersaturated in SBF 70P + MOPS solution than in the supersaturated one. The dissolution process accelerated to the moment of HAp formation on the 11th day of exposure in SBF 70P + MOPS (Figure 7).

**Figure 8:**
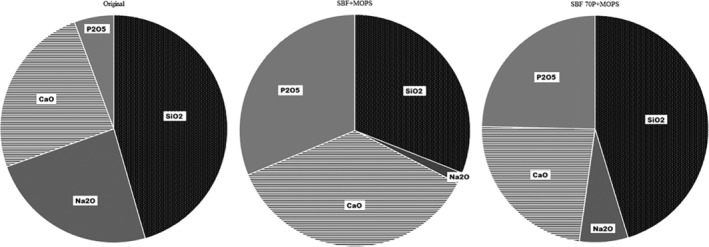
Ratios of scaffold components (% of oxides) in original scaffold, after 15 days of interaction in SBF + MOPS and after 13 days in SBF 70P + MOPS

The WD‐XRF analysis of the residual glass–ceramic scaffold exposed to SBF + MOPS showed a significant release of Na^+^ ions (recalculated as Na_2_O). After 8 hr, 50 wt% of its original content was released, which confirmed the incongruent dissolution of the glass–ceramic. Conversely, the concentration of (PO_4_)^3−^ ions (recalculated as P_2_O_5_) in the scaffold strongly increased. The same trend was observed for the concentrations of Si and Ca; SiO_2_ being released into solution and CaO increasing in the scaffold. The content of CaO increased due to the precipitation of the new Ca‐P phase (Table 3a, Figure 8). At the beginning of the exposure, these results are ostensibly inconsistent with those of the leachate analyses (Figure 2) due to the higher rate of Ca‐P phase precipitation than of Ca^2+^ ions release into SBF + MOPS. Later, the rates of both processes became balanced.

**Table 3:** Chemical composition of the original scaffold before and after exposure to (a) SBF + MOPS (wt%; WD‐XRF) and (b) SBF 70P + MOPS (wt%; WD‐XRF), normalized to 100% and CaO/P_2_O_5_ ratio changes

Time (days)	SiO_2_	CaO	Na_2_O	P_2_O_5_	CaO/P_2_O_5_ ratio^a^
*(a) SBF + MOPS (wt%; WD‐XRF)*					
Origin	45.1	24.5	23.8	5.6	4.4
0.3	47.4	24.7	12.7	13.1	1.9
1	45.8	24.4	12.9	14.3	1.7
3	43.2	26.1	8.8	19.6	1.3
7	35.1	30.8	3.1	28.6	1.1
11	32.8	31.9	2.3	29.4	1.1
15	30.0	35.1	1.5	30.3	1.2
*(b) SBF 70P + MOPS (wt%; WD‐XRF)*					
Origin	45.1	24.5	23.8	5.6	4.4
0.3	48.6	23.9	17.4	9.5	2.5
1	48.4	22.9	15.5	11.5	2.0
3	48.7	24.4	12.4	14.5	1.6
7	50.0	22.1	14.0	13.1	1.7
13	42.9	26.8	11.2	17.7	1.5
11	44.6	22.8	6.8	24.3	0.9
15^b^	–	–	–	–	–

a Hydroxyapatite—Ca_10_(PO_4_)_6_(OH)_2_: theoretical CaO/P_2_O_5_ ratio = 1.32.

b Day 15: not analyzed—low amount of sample.

When the concentration of (PO_4_)^3−^ ions was insufficient in SBF 70P + MOPS to precipitate, all of the above‐described processes were slower. The concentration of Na^+^ ions decreased to 70 wt% of their original value in the glass–ceramic scaffold after 8 hr of exposure. Moreover, apart from the incongruent dissolution of the scaffold (alkalis diffusion), a large part of the glass–ceramic material was totally dissolved. By the end of the test, more than 75 wt% of the original scaffold weight had been lost. The formation of the Ca‐P phase was negligible (Table 3b, Figure 8), as confirmed by XRD (Figure 7).

**Figure 9:**
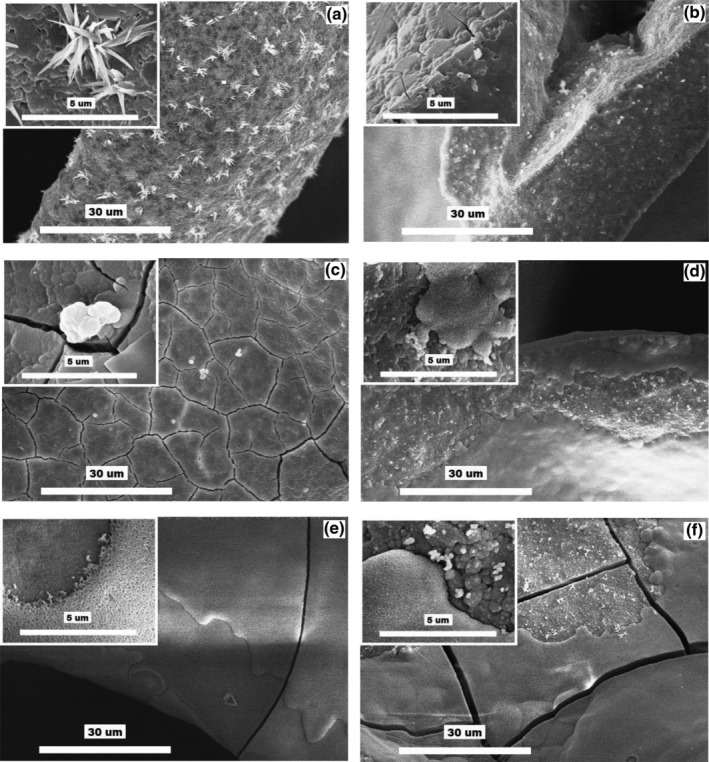
SEM images at two different magnifications of glass–ceramic scaffold: (a) before exposure (combeite = the small tabular crystals, structurally isomorphic buchwaldite, and CaO∙SiO_2_ phases = the needle‐like crystals); (b–f) after 0.3, 1, 3, 7, and 15 days of interactions with SBF + MOPS, respectively

The interactions of the glass–ceramic scaffold with both solutions during the in vitro tests were also documented by SEM/EDS images (Figure 9). The material surface immersed in SBF + MOPS significantly changed during the first 8 hr. The needle‐like crystals of minor crystalline phases completely disappeared. After 24 hr, a thin layer of the very fine globule‐shaped crystals covered the material surface. The new layers of HAp gradually covered the glass–ceramic scaffold, completely covering it by the end of the test (Day 15).

The specific surface of the glass–ceramic scaffold (measured by BET) strongly increased from its original value of 2.6 to 32.2 m^2^.g^−1^ (13×) by the end of the third day (Table 4).

**Table 4:** Specific surface values for scaffold immersed in both solution (m^2^ g^−1^)

Time (days)	SBF + MOPS	SBF 70P + MOPS^a^
0	2.6	2.6
0.3	8.8	7.2
1	9.9	6.7
3	32.8	10.1
7	35.7	–
11	26.9	–
15	32.3	–

a Days 7–15: not analyzed—low amount of sample.

**Figure 10:**
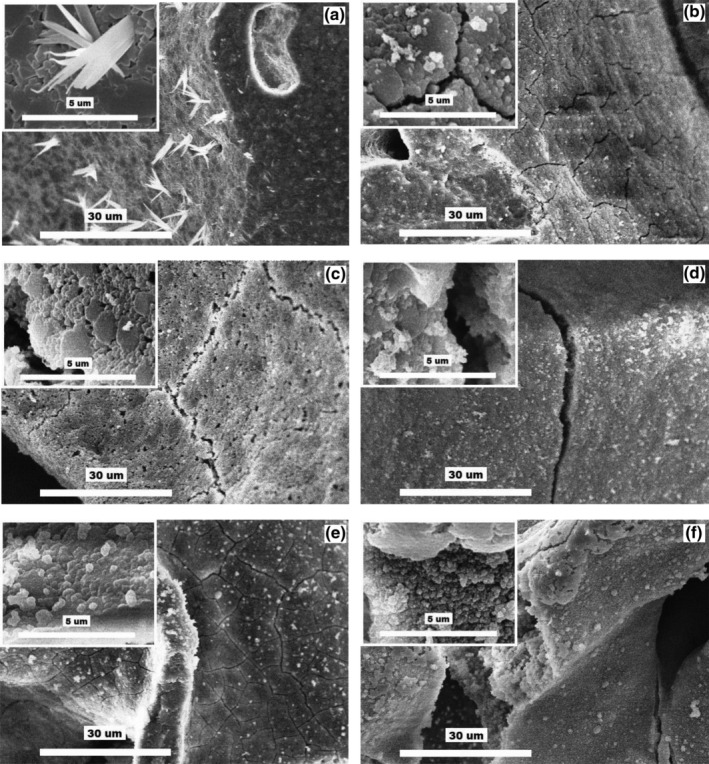
SEM images at two different magnifications of glass–ceramic scaffold: (a) before exposure (combeite = the small tabular crystals, structurally isomorphic buchwaldite, and CaO∙SiO_2_ phases = the needle‐like crystals); (b–f) after 0.3, 1, 3, 7, and 13 days of interactions with SBF 70P + MOPS, respectively

In the SBF 70P + MOPS solution, the glass–ceramic scaffold clearly dissolved. Individual globules of the HAp phase appeared only sporadically on the cracked surface of the scaffold (Figure 10). By the end of Day 3, the specific surface had increased by a factor of 5.

## 4 DISCUSSION

Analysis of the SBF + MOPS leachates (Ca^2+^, (PO_4_)^3−^ and Si^IV^ concentrations) together with analysis of the scaffold (XRD, XRF) confirmed the intensive dissolution of the scaffold crystalline phase (Combeite), which is in agreement with our previous findings regarding the TRIS and HEPES buffers (Rohanová et al., 2018). The MOPS buffer (as well as the TRIS and HEPES buffers) did not have the capacity to maintain a neutral environment in SBF due to the very quick diffusion of Na^+^ ions from the scaffold (during the first 8 hr, 50% of the original amount was released). Simultaneously with the diffusion process, the total dissolution of the glass–ceramic occurred (based on the released Si).^1^ By the end of Day 3, 54 wt% of the scaffold material had dissolved. The above‐mentioned processes resulted in an increase in the saturation of the already supersaturated metastable SBF + MOPS. Consequently, a crystalline form of HAp was recorded after 3 days. The everyday exchange of the SBF + MOPS solution by a fresh one supplied a sufficient amount of Ca^2+^ and (PO_4_)^3−^ ions to maintain a constant HAp growth rate during the test. The newly formed HAp layer totally covered the scaffold surface. The HAp protective layer slowed down scaffold dissolution after Day 5 of immersion in SBF + MOPS (Figure 4). After Day 7, both processes, alkali diffusion and glass–ceramic dissolution, stopped and the rate of the HAp precipitation stabilized. From this point, the growth of the newly formed HAp became dependent on the SBF solution (supersaturation) and not on the quality of the original material, as we also observed in the cases of the ß‐TCP and HA interactions (Horkavcová, Zítková, Rohanová, Helebrant, & Cílová, 2010).

This phenomenon was confirmed by the in vitro test in undersaturated SBF 70P + MOPS. Ca‐P phase precipitation was expected when the concentration of (PO_4_)^3−^ ions strongly decreased at the beginning of the test (until 24 hr). However, the concentration stabilized around its original value from Day 2 until the end of the test. Without any doubt, the phosphorus in the glass phase of the scaffold was not released quickly enough. Therefore, the concentration of (PO_4_)^3−^ ions in SBF 70P + MOPS did not reach the supersaturated state needed for HAp precipitation. Due to the very low rate of HAp formation (its presence was only noticed after Day 11) and the insufficient protective behavior of the layer (Figure 4), the glass–ceramic scaffold had nearly completely dissolved by the end of Day 13. Moreover, the specific surface (measured by BET) of the HAp layers was four times higher in SBF + MOPS (36.9 m^2^ g^−1^) than in SBF 70P + MOPS (10.0 m^2^ g^−1^) after Day 3 (the following days were not possible to compare due to the very low remaining amount of scaffold exposed in SBF 70P + MOPS). Together, all of these processes confirm the role of SBF solution supersaturation, which, in the case of a highly soluble material, is strengthened by an aggressive buffer.

## 5 CONCLUSION

MOPS buffer accelerates the dissolution of the scaffold crystalline phase (Combeite), thereby supporting HAp crystallization. The rapid formation of a HAp layer with a large specific surface protects the scaffold against further material dissolution. Phosphorus released from the glass phase of the scaffold does not significantly contribute to HAp formation.

Thus far, we have shown that each of the tested buffers (TRIS, HEPES, and MOPS) affects the kinetics of the dissolution of glass–ceramics materials in a specific way. Our next work will be focused on the TES and BES buffers, the last untested buffers from the family of Good's buffers.

## Supporting information


**Appendix S1**: Supporting information

## Endnote

1 Due to a simplification, we assume that Si did not form any new phase.
