# Supplementary material for: Interaction of MOPS buffer with glass–ceramic scaffold: Effect of (PO4)3− ions in SBF on kinetics and morphology of formatted hydroxyapatite
Source: J Biomed Mater Res B Appl Biomater. 2019 Dec 16;108(5):1888–96. doi: 10.1002/jbm.b.34530 (PMC7217194; doi:10.1002/jbm.b.34530)
Supplement: Supplementary file 1 — Appendix S1: Supporting information [file JBM-108-1888-s001.docx]

**Goals of this work**

ISO standard 23317:2014 uses the TRIS buffer to maintain a neutral pH in SBF. In our previous papers, we reported the use of TRIS (*TRIS buffer in simulated body fluid distorts the assessment of glass-ceramic scaffold bioactivity. Acta Biomater 2011;7:2623-2630*) and HEPES (*Interaction of HEPES buffer with glass-ceramic scaffold: Can HEPES replace TRIS in SBF?. J Biomed Mater Res part B: Appl Biomater 2018;106B:143-152.*) buffers for the *in vitro* testing of an inorganic glass-ceramic scaffold.

However, the interaction of a tested glass-ceramic material with TRIS can produce false positive results. In fact, the presence of the buffers in SBF more than doubled the rate of glass-ceramic dissolution and enhanced HAp crystallization.

Our new manuscript solve the MOPS (3-(*N*-*morpholino) propanesulfonic acid*) buffer from the group of Good´s buffers. We believe that our results will help to better understanding the interactions of highly reactive biomaterials with SBF.
